# Imaging aspects of the central nervous system in dengue

**DOI:** 10.1590/0100-3984.2017.50.6e2

**Published:** 2017

**Authors:** Nina Ventura

**Affiliations:** 1 MD, Radiologist at DASA and at the Instituto Estadual do Cérebro Paulo Niemeyer, Rio de Janeiro, RJ, Professor of Radiology at the Universidade Federal Fluminense (UFF), Niterói, RJ, Brazil. E-mail: niventuraa@gmail.com

Dengue is an infectious disease caused by an RNA virus of the family Flaviviridae (genus *Flavivirus*); there are four dengue virus serotypes (DEN1 through DEN4), which are transmitted to humans mainly by the *Aedes aegypti* mosquito^(1)^. In the last 50 years, the incidence of dengue has increased 30-fold, and the geographic distribution of the disease has expanded to include new countries. It is estimated that 50 million dengue infections occur annually and that approximately 2.5 billion people live in countries where the disease is endemic^(1)^.

The main clinical manifestations are myalgia, arthralgia, skin rash, vomiting, and nausea; the most common complications are shock and dengue hemorrhagic fever^(2)^. The central neurological manifestations are found in severe cases and are generally subdivided into three categories^(3,4)^: encephalopathy secondary to systemic involvement, due to shock, hypoxia, hyponatremia, renal failure, or hepatic failure; encephalitis due to direct invasion by the virus; and immune-mediated demyelination or vasculitis. It is believed that serotypes DEN2 and DEN3 are directly related to neurological impairment^(4)^.

The diagnostic criteria for dengue encephalitis include clinical data consistent with febrile encephalopathy and positivity for immunoglobulin M antibodies or positive polymerase chain reaction results for dengue in the blood or cerebrospinal fluid, as well as exclusion of other potential causes of viral encephalitis^(4)^.

Although magnetic resonance imaging (MRI) is the main method of assessing the neurological damage caused by dengue virus, there have been few studies describing the imaging aspects of such damage. The main changes related to encephalitis are fairly nonspecific and include bilateral hyperintense signals in T2-weighted and fluid-attenuated inversion recovery (FLAIR) sequences, especially in the basal ganglia and thalamus. Foci of restricted diffusion and discrete areas of contrast enhancement can also be seen. Other potentially affected regions are the hippocampus, the bridge, the cerebellum, and the corpus callosum^(5-7)^. Cerebral microhemorrhages can be seen in patients with acute hemorrhagic encephalitis and are best demonstrated in gradient sequences^(8)^. Alterations of the white matter, especially the periventricular white matter, can be seen in immune-mediated demyelinating processes^(8)^. In rare cases, the presentation of vasculitis secondary to dengue infection includes focal infarcts^(9)^. These findings are not specific for dengue virus infection. The differential diagnosis should include other types of viral encephalitis and acute disseminated encephalomyelitis (ADEM). In addition, patients with encephalitis, especially those with dengue encephalopathy, can present normal MRI findings^(8)^.

In the previous issue of **Radiologia Brasileira**, Jugpal et al.^(10)^ presented a study involving nine patients with neurological manifestations of dengue fever who were submitted to MRI of the brain. The MRI findings corroborated those of other authors, the main alterations being hyperintense signals in T2-weighted and FLAIR sequences, together with restricted diffusion and hemorrhagic foci, predominantly in the basal ganglia and thalamus. Their article also reviewed the imaging alterations seen in the main differential diagnoses, which include Japanese encephalitis, herpes simplex encephalitis, and ADEM, noting the findings that favor each diagnosis.

There is no specific treatment for dengue. Nevertheless, knowledge of the main MRI findings in patients with neurological manifestations of dengue fever is essential for early recognition of the severe form of the disease, facilitating symptomatic treatment and preventing complications.
